# Effect of Plasma Gel on Viability, Proliferation and Migration of Dermal Human Fibroblasts In Vitro

**DOI:** 10.3390/gels12080713

**Published:** 2026-08-12

**Authors:** Camila Slompo, Emanuelle Pangoni de Carvalho, Thayna Kathllen da Silva, Byron Israel Proaño Roldan, Juliana Sanches Trevizol, Elizandra Paccola Moretto de Almeida, Adriano de Souza Pessoa, Thais Francini Garbieri, Mariana Liessa Rovis Sanches, Rodrigo Cardoso de Oliveira, Marília Afonso Rabelo Buzalaf

**Affiliations:** 1 Department of Biological Sciences, Bauru School of Dentistry, University of São Paulo, Al. Dr. Octávio Pinheiro Brisolla, 9-75, Bauru 17012-901, São Paulo, Brazil; camilaslompo@usp.br (C.S.); emanuellecarvalho@usp.br (E.P.d.C.); thayna.kathllen14@usp.br (T.K.d.S.); byronipr@usp.br (B.I.P.R.); julianatrevizol@usp.br (J.S.T.); elizandra.moretto@gmail.com (E.P.M.d.A.); adrianospessoa@usp.br (A.d.S.P.); tfgarbieri@usp.br (T.F.G.); marianasanches@usp.br (M.L.R.S.); rodrigocardoso@usp.br (R.C.d.O.); 2 Hospital for Rehabilitation of Craniofacial Anomalies, University of São Paulo (HRAC/USP), Bauru 17012-902, São Paulo, Brazil

**Keywords:** platelet rich fibrin, collagen, fibroblasts, plasma gel

## Abstract

This study evaluated plasma gel preparation protocols with potential to yield more biocompatible materials for facial aesthetic applications. Human dermal fibroblasts were cultured in medium supplemented with 20% plasma gel obtained using four protocols: G1, centrifugation at 580 g for 8 min in tubes with anticoagulant followed by activation with 10% calcium chloride; G2, centrifugation at 580 g for 8 min in tubes with anticoagulant without activation; G3, centrifugation at 210 g for 8 min in tubes without anticoagulant with activation; and G4, centrifugation at 210 g for 8 min in tubes without anticoagulant and without activation. All protocols involved heating plasma at 76 °C for 12 min to produce gels. Cell viability was assessed using MTT and Crystal Violet assays over 72 h. Cell morphology and fibrin formation were analyzed by hematoxylin/eosin staining. Cell migration was evaluated using a wound healing assay. RT-qPCR analysis of COL1A1 and FN1 was performed after 24, 48, and 72 h. Fibroblasts cultured with plasma gel from G3 and G4 showed significantly higher viability compared with the control group. G1 and G2 exhibited increased fibrin formation and reduced cell density. Slower wound closure was observed in G2, which was consistent with increased late-stage expression of COL1A1 and FN1. In the conditions evaluated, low-speed centrifugation and the use of tubes without anticoagulant improved plasma gel biocompatibility. Lack of activation after high-speed centrifugation was associated with slower wound closure.

## 1. Introduction

Currently, the demand for procedures for dermal filling and collagen stimulation has grown exponentially. The most widely used product for dermal augmentation is hyaluronic acid [1]. With the increase in patient demands, new materials and combinations thereof have emerged. Nowadays, there are several treatment options with different purposes and costs, which have broadened access of the population to these procedures [2].

Non-invasive aesthetic facial treatments range from subtle improvements in fine wrinkles and skin quality, to more pronounced changes in facial proportions, depending on the patient’s needs and complaints. There are several possible procedures, employing distinct products and techniques, such as filling with hyaluronic acid fillers, application of botulinum toxin, skinboosters, microneedling, and collagen bio-stimulators, among others [2,3].

As biodegradable substances, these exogenous injectable products may have distinct disadvantages. They may cause transient effects such as persistent erythema, swelling and encapsulation, granuloma formation, and sometimes even chronic or late infections [2]. Consequently, professionals have sought autologous sources of fillers and tissue biostimulators. In addition, patients also seek increasingly natural treatments with less risk of complications and often do not want to inject synthetic materials. In this sense, autologous platelet concentrates have gained increasing prominence, and their use has become increasingly common [1,2,4]. They have long been used in dental surgeries for wound healing and regeneration in different areas and, more recently, have been applied in facial aesthetics [4]. The greatest advantage of this technique is the possibility of using the patient’s own blood for treatment, therefore providing a more biocompatible option than commercial fillers and collagen biostimulators [4,5]. Among these products are platelet-rich plasma (PRP), injectable platelet-rich fibrin (i-PRF) and plasma gel fillers [2,6].

PRP is a platelet concentrate obtained from the patient’s own blood [2,7]. It is prepared through several steps, including centrifuging the patient’s own blood in tubes with anticoagulants (ACs), to obtain a platelet-rich concentrate that can be used for different purposes [2,4,7]. In addition to the need for ACs, several centrifugation steps are required to obtain PRP. Furthermore, the focus of PRP is on the advantages of platelets and their growth factors, which support tissue regeneration in different medical fields [4,7,8,9].

However, in an effort to minimize the risk of contamination and eliminate the use of ACs (which are known inhibitors of tissue regeneration), a new material, known as platelet-rich fibrin (PRF), was introduced as the first platelet concentrate without ACs. Initially, centrifugation was performed in glass tubes, leading to physiological activation of the coagulation cascade and thus the formation of a fibrin clot enriched with platelets and leukocytes. The fibrin clot allows the sustained release of growth factors, representing an advantage over PRP [4,10]. In 2014, a liquid platelet concentrate was developed based on the concept that centrifugation at a lower speed results in a greater quantity of leukocytes, platelets and growth factors. Plastic tubes were employed to maintain the product in a liquid state for a longer time before coagulation, thereby allowing injection and use in facial aesthetics. This product was called injectable-platelet-rich fibrin (i-PRF) and is obtained by a single centrifugation step in a plastic white-top tube without any type of additive or AC and without biochemical manipulation of the blood [11,12,13].

As with PRF, the protocols for obtaining i-PRF are also variable. Shashank et al. (2021) [14] employed 800 revolutions per minute (RPM) (78 g) for 4 min, whereas others recommended centrifugation at 700 RPM (60 g) for 3 min [15] or at 200 g for 10 min and 150 g for 5 min for surgical and aesthetical purposes, which are called “Surgical Fibrin” and “Aesthetic Fibrin”, respectively, following the Low-Speed Centrifugation Concept (LSCC) [12]. It has been reported that reducing the centrifugation speed and time results in greater preservation of cellular components (platelets and leucocytes), thereby increasing growth factor release [16].

In search of a blood-derived filler, the plasma gel filler was developed. This biomaterial combines thermal denaturation of plasma with cross-linking of autologous fibrin [17]. It is based on collecting a small volume of the patient’s blood, which is subsequently centrifuged and activated to obtain an autologous clot enriched with proteins and growth factors [17,18]. This gel-like formulation provides long-term volume stability while delivering bioactive molecules. After heating and cooling, the product is enriched with liquid PRF, thereby improving tissue regeneration. The regenerative potential of this autologous protein-based gel was demonstrated by increased cell proliferation and stimulation of extracellular matrix (ECM) protein synthesis [17]. In this technique, blood is collected in blue-capped tubes containing AC, centrifuged at 580 g for 8 min, and platelet-poor plasma (PPP) is incubated at 76° C for 12 min before being mixed with PRP, previously activated with PRGF-Endoret Activator (10% calcium chloride—CaCl_2_) [17].

A wide variety of centrifugation protocols have been described. Some protocols employ double centrifugation, whereas others use a single centrifugation step [17,19,20], which is sufficient to separate PPP and PRP [21,22] and facilitates clinical application by simplifying the procedure and reducing preparation time. In both approaches, different centrifugation times and forces have been reported. Dohan Ehrenfest and collaborators [23] highlighted the difficulty of standardizing centrifugation protocols, as these parameters are often empirically defined and inconsistently interpreted. In most studies, platelet concentrates were not adequately described or characterized, particularly regarding their cellular content, with many authors considering all platelet concentrates to be equivalent regardless of composition.

It is important to note that heating PPP at 76 °C to obtain plasma gel denatures growth factors, rendering the material inert, without biostimulatory potential. To overcome this limitation, the F2 fraction, rich plasma (RP), which is rich in growth factors, is added after gel formation [17]. Moreover, following the LSCC, a reduction in relative centrifugal force (RCF) increases cellular and growth factor concentration, thereby enhancing the biostimulatory potential of the material. In the present study, we propose reducing the RCF to 210 g and using white-capped tubes without ACs for blood collection, as anticoagulants impair the wound healing process [16]. Additionally, we evaluated the need for biomaterial activation with CaCl_2_. The response of human dermal fibroblasts to plasma gels prepared using these distinct thermal protocols was evaluated in terms of viability, proliferation, and migration, aiming to identify the most suitable protocol for facial aesthetic applications.

## 2. Results and Discussion

### 2.1. Viability Assays–MTT, Crystal Violet and Neutral Red

**Figure 1:**
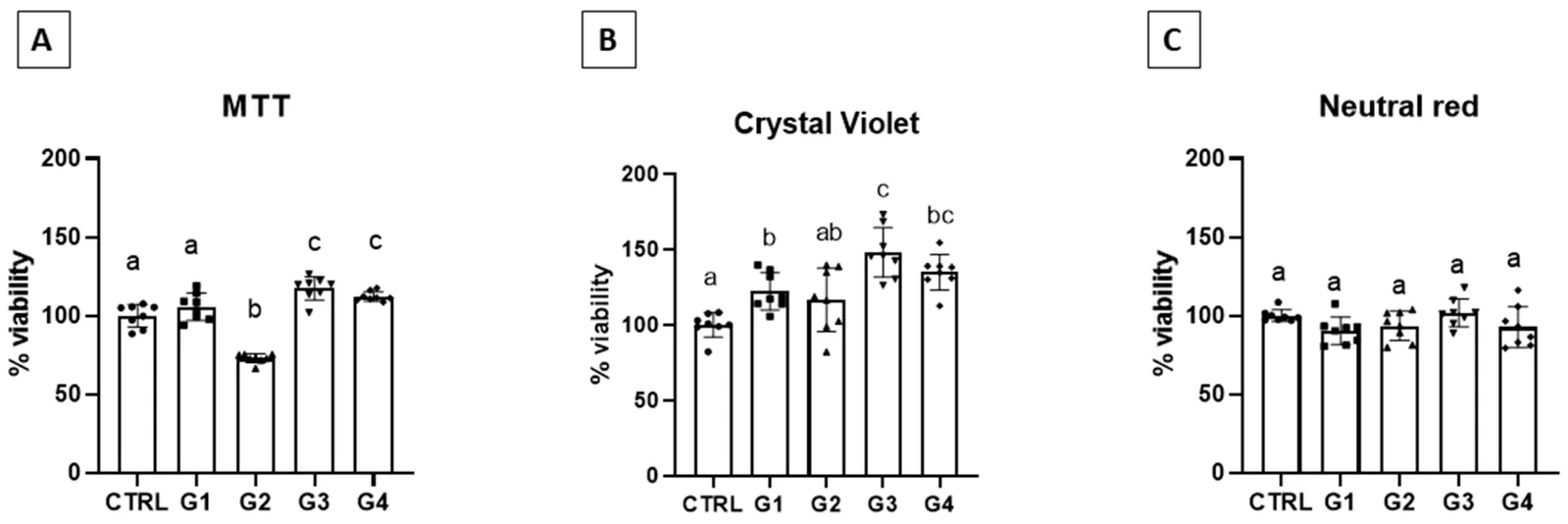
Viability Assays: (**A**) MTT, (**B**) Crystal Violet and (**C**) Neutral Red assay. G1: 580 g + CaCl_2_; G2: 580 g; G3: 210 g + CaCl_2_; G4: 210 g. One-way ANOVA with the Tukey post hoc test was used. Values are expressed as mean (%) ± standard deviation of two independent experiments performed with *n* = 4. For each assay, distinct letters indicate significant differences between groups (ANOVA and Tukey’s test, *p* < 0.05).

As shown in Figure 1, cell viability and proliferation were evaluated after 72 h using complementary assays that assess distinct biological parameters. In the MTT assay, which reflects mitochondrial metabolic activity, a significant increase in cell viability was observed in Groups G3 and G4 compared to the control group (Figure 1A). In contrast, Group G2 showed significantly reduced metabolic activity compared with all the other groups, indicating a less favorable cellular response under this condition. There were no significant differences between the control group and G1 or between G3 and G4.

In the Crystal Violet assay, which quantifies the total number of adherent cells, Groups G1, G3 and G4 showed a significant increase in cell density compared to the control group after 72 h (Figure 1B). Although Group G2 presented higher mean absorbance values, this increase was not statistically significant when compared to the control group. G3 showed a significant increase in cell density when compared with all the other groups, except G4. There was no significant difference in cell density between G1 and G2 or between G3 and G4. These results suggest a partial discrepancy between metabolic activity and adherent cell population among the experimental conditions.

The combined analysis of the MTT and Crystal Violet assays revealed a variation between metabolic activity and the number of adherent cells in Group G2 (Figure 1A,B). Based on these findings, the Neutral Red assay was incorporated to evaluate lysosomal integrity and, consequently, confirm cell viability from an additional functional perspective.

In the Neutral Red assay, which assesses cell viability based on lysosomal uptake capacity, no significant differences were observed between any of the groups after 72 h (Figure 1C) in FBH CK 001 fibroblast cells. This suggests that despite the variations observed in metabolism (MTT) and adhesion/proliferation (Crystal Violet), the cells maintained their structural integrity, even in Group G2.

### 2.2. Morphological Analysis

**Figure 2:**
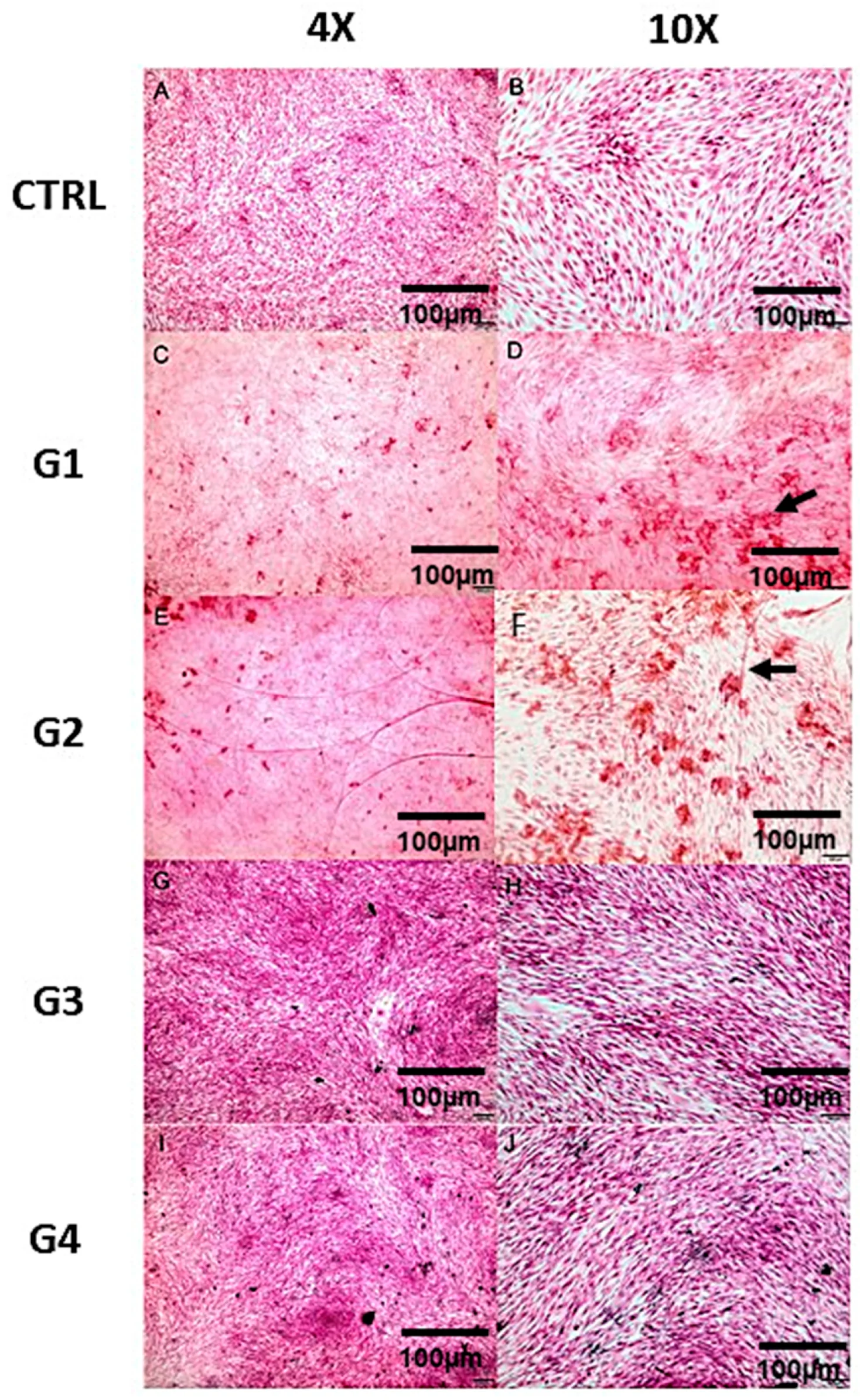
Cell Morphological Analysis by Hematoxylin and Eosin staining under an Olympus SC30 microscope after treatment of FBH-CK001 cells for 72 h with the different plasma gel preparations or not (control); 4× and 10× magnification, scale bar 100 μm. G1: 580 g + CaCl_2_; G2: 580 g, G3: 210 g + CaCl_2_; G4: 210 g. The arrows indicate the presence of fibrin.

Qualitative morphological analysis was performed by hematoxylin-eosin (HE) staining after 72 h of treatment with plasma gel. Compared with the control group, there was an increase in the number of FBH-CK001 cells after treatment with plasma gels, particularly in groups 3 (G, H) and 4 (I, J) compared with the control group (A, B). The presence of a large amount of fibrin in groups 1 (C, D) and 2 (E, F) hindered cell visualization. Regarding cell morphology, cells in these groups appeared smaller and exhibited slight morphological alterations, whereas in the other groups, cell morphology was preserved, with a similar pattern among groups, differing mainly in cell number (Figure 2).

### 2.3. Wound Healing Assay

**Figure 3:**
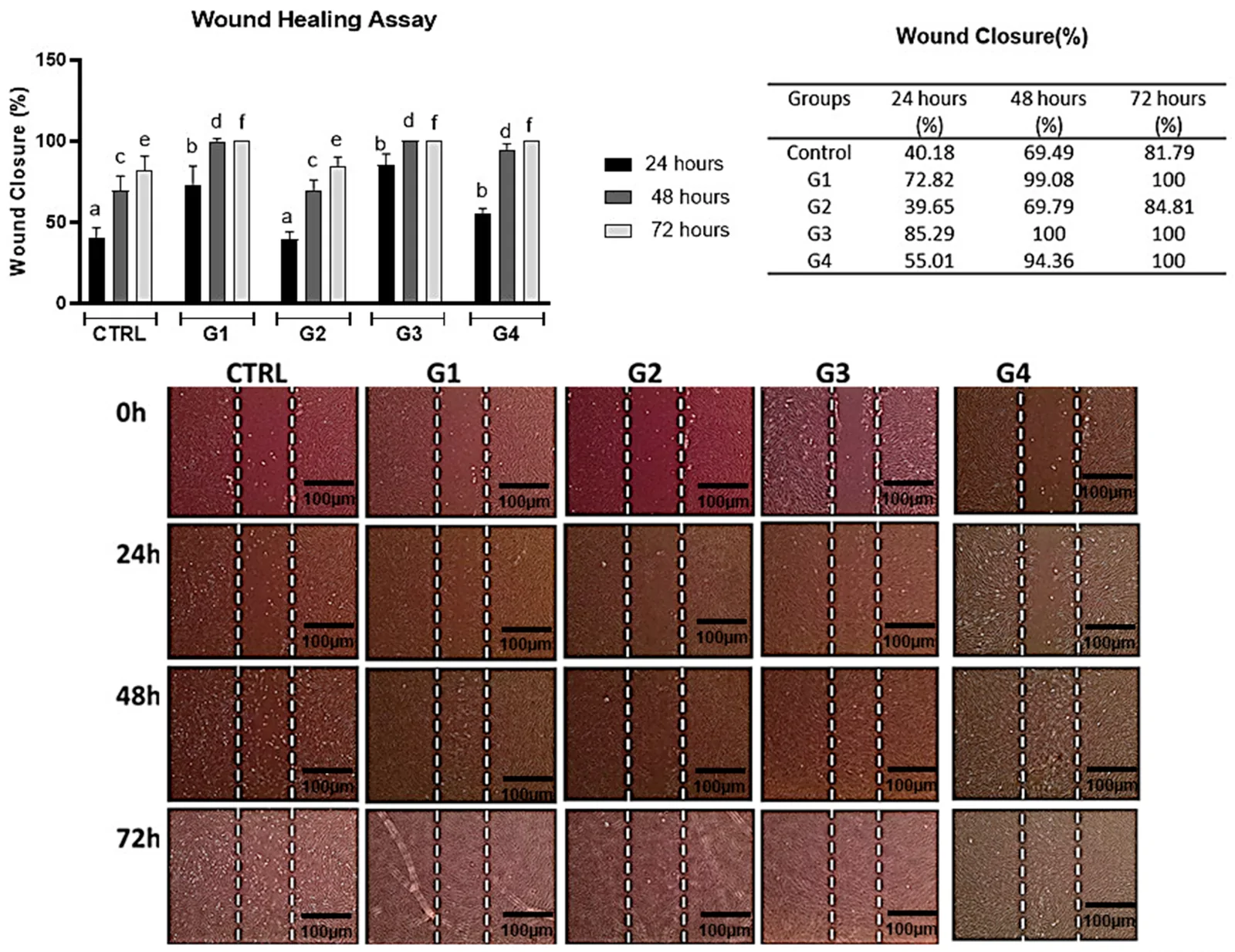
Wound Healing Assay. Table with the closing percentages for each group at 24, 48 and 72 h. G1: 580 g + CaCl_2_; G2: 580 g, G3: 210 g + CaCl_2_; G4: 210 g. One-way ANOVA with the Tukey post hoc test was used (*p* < 0.0001). Values are expressed as mean (%) ± standard deviation of two independent experiments performed with *n* = 4. The wound healing was analyzed by ImageJ software (Version 13.0.6). Letters above the bars indicate significant differences compared to the control group within each specific time. At time 0, we observed only the cleft in the cells; then, at 24 h, moderate migration of fibroblasts, mainly in group G3, while at 48 h, almost total migration of fibroblasts was observed, except for group G2. Finally, at 72 h, total migration/healing of fibroblasts in the cleft was observed, except for group G2.

The wound healing assay demonstrated effective migration in all groups. Time 0 shows only the initial slit for groups CTRL, G1, G2, G3, as well as group G4. The groups G1, G3, and G4 achieved complete wound closure after 72 h, unlike the control group and G2. Group G3 exhibited the fastest wound closure, which was complete after 48 h, followed by G1 and G4, which showed nearly complete (~100%) closure at 48 h. After 72 h, the control group and G2 did not achieve complete closure and displayed similar migration patterns. In G1 and G2, the presence of fibrin was observed, particularly at 72 h. Activation with calcium chloride enhanced fibroblast migration, especially when centrifugation was performed at 580 g in tubes with AC (G1) (Figure 3).

### 2.4. RT-qPCR Assay

**Figure 4:**
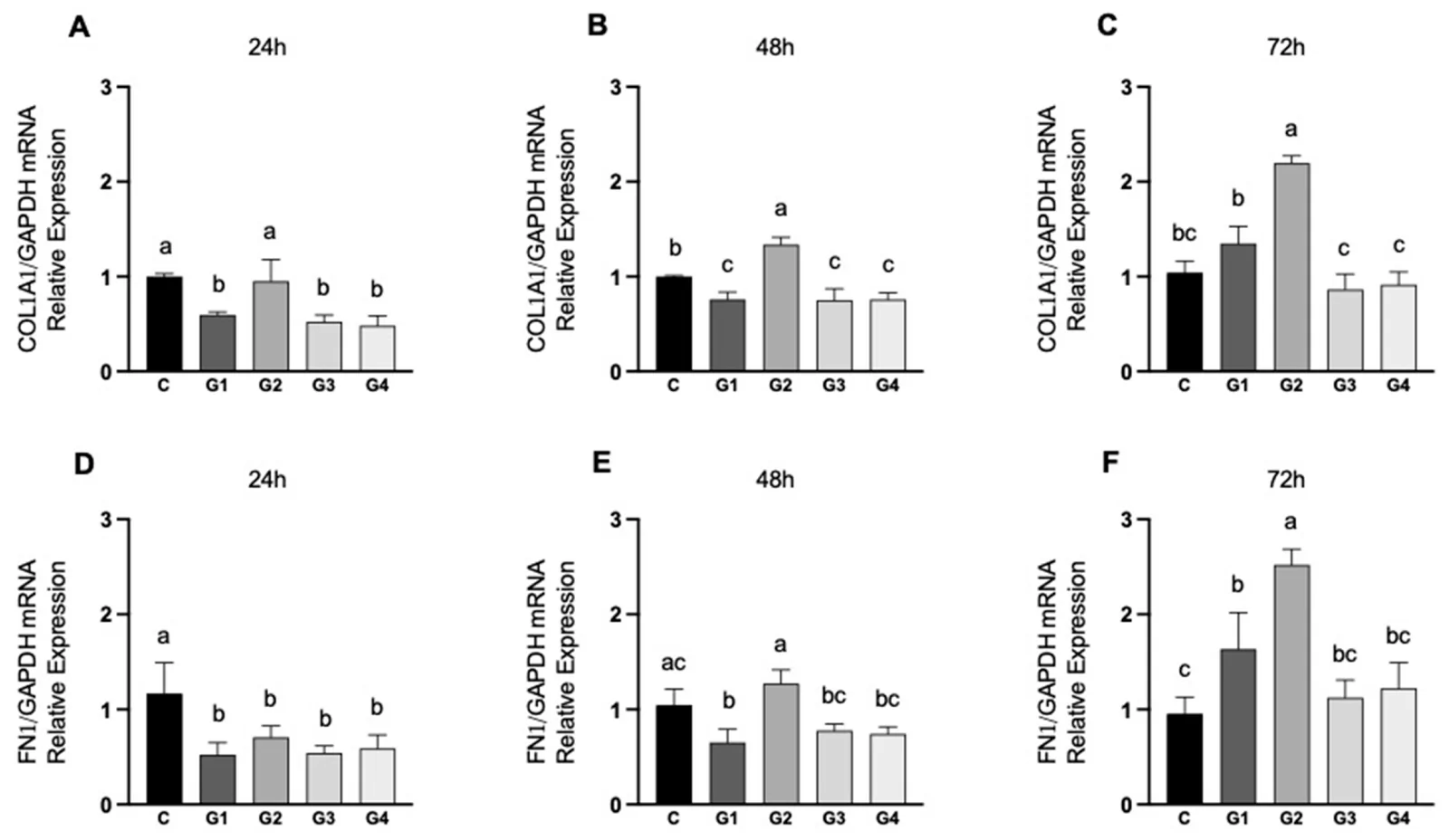
Relative mRNA expression of COL1A1 and FN1 in relation to the reference gene (GAPDH) assessed by RT-qPCR after 24, 48, and 72 h of treatment. COL1A1 gene expression is shown at 24 (**A**), 48 (**B**), and 72 h (**C**), whereas FN1 gene expression is shown at 24 (**D**), 48 (**E**), and 72 h (**F**). For each time point, distinct letters indicate significant differences between groups (ANOVA and Tukey’s test, *p* < 0.05).

In G1, G3 and G4, at 24 h, COL1A1 mRNA expression was significantly lower compared with the control group and G2, which did not significantly differ from each other (Figure 4A). At 48 h, G1, G3, and G4 continued to exhibit significantly lower COL1A1 expression than the control group and G2, which presented the highest expression, significantly differing from all other groups (Figure 4B). At 72 h, COL1A1 mRNA expression was markedly increased in G2, being significantly higher than in all other groups. G1, G3 and G4 did not significantly differ from the control group (Figure 4C).

Regarding FN1 mRNA expression, at 24 h, all the experimental groups presented similar expression levels, which were significantly lower than those of the control group (Figure 4D). At 48 h, G1 still showed significantly lower expression than the control group, but did not significantly differ from G3 and G4, which also did not differ from the control. G2 did not significantly differ from the control group but exhibited expression levels significantly higher than G1, G3, and G4 (Figure 4E). At 72 h, the highest FN1 expression was observed in G2, which significantly differed from all other groups. G1 also presented significantly higher expression levels than the control group but did not significantly differ from G3 and G4. These, in turn, did not significantly differ from the control (Figure 4F).

### 2.5. Discussion

In the present study, the cytotoxicity of plasma gel prepared with two different RCFs, with or without activation with CaCl_2_, and from blood collected in tubes with or without AC, was investigated in vitro using human dermal fibroblasts. To the best of our knowledge, this is the first study to apply the LSCC to plasma gel preparation, aiming to contribute to the standardization of centrifugation protocols for facial aesthetic practice based on scientific evidence.

It has been shown that high-speed centrifugation results in the loss of leukocytes and growth factors, which are forced to the bottom of the centrifugation tubes due to excessive RCF [11]. For this reason, LSCC has been proposed to improve the quantity of cells and growth factors retained within PRF scaffolds [11]. In the present study, two centrifugation protocols for plasma gel preparation were compared. The first was the protocol proposed by Anitua et al. [17], which employs an RCF of 580 g for 8 min. The second protocol was previously described for PRF-based matrices and developed by Kobayashi et al. (2016), employing an RCF of 208 g for 8 min (A-PRF+) [16]. This approach yields a more porous PRF matrix, enriched with neutrophilic granulocytes, and results in increased cellular content and growth factor release [12,17].

The present results demonstrated that plasma gels favored the proliferation and migration of human dermal fibroblasts, as evidenced by viability assays (MTT and Crystal) and the wound healing assay over a 72 h period. Notably, Groups 3 and 4, in which blood was collected in tubes without AC and centrifuged at a reduced RCF (210 g), exhibited the most favorable outcomes regarding viability, proliferation, and migration after 72 h of treatment. The concept of removing ACs originated from the seminal work of Choukroun et al. (2001) [10], which introduced PRF as a second-generation platelet concentrate [10]. Thus, the superior performance of G3 and G4 observed in the present study is consistent with two established PRF principles: LSCC [11,12,16] and the benefits of eliminating ACs from blood collecting tubes [2,10]. In the Crystal Violet assay, however, a false-positive effect was observed in G2, likely due to excess fibrin staining together with cells, which artificially increased absorbance values.

The combined analysis of MTT, Crystal Violet and Neutral Red assays indicates that plasma gels obtained under low-speed centrifugation conditions (210 g) and in the absence of anticoagulants (G3 and G4) promote a more favorable cellular response, particularly at the metabolic level. The MTT assay revealed a significant increase in mitochondrial activity in these groups, suggesting enhanced cellular functionality and proliferation. In parallel, the Crystal Violet assay demonstrated a higher adherent cell population in G3 and G4, supporting increased cell attachment and growth on fibrin-rich matrices. These findings are consistent with the concept that lower RCF protocols favor the retention of platelets, leukocytes, and growth factors within the fibrin network, resulting in a more bioactive scaffold capable of supporting fibroblast activity [6,12].

In contrast, Group G2, prepared under higher centrifugal force (580 g) in the presence of ACs and without activation (10% CaCl_2_), exhibited reduced metabolic activity and no significant increase in lysosomal viability, as demonstrated by MTT and Neutral Red assays (Figure 1A,C). Although a non-significant increase in Crystal Violet (Figure 1B) staining was observed, this finding is likely influenced by fibrin-related dye retention rather than an increase in viable adherent cells.

Importantly, the absence of statistically significant differences in the Neutral Red assay across all groups suggests that lysosomal integrity remains relatively preserved under all experimental conditions, while mitochondrial activity appears to be more sensitive to variations in plasma processing. Collectively, these results reinforce that both centrifugation parameters and blood collection conditions critically influence the biological performance of plasma-derived gels, with low-speed, anticoagulant-free preparations yielding a more favorable microenvironment for fibroblast function.

The study by Fedyakova et al. (2019) [19] also employed Anitua’s centrifugation and plasma gel preparation protocol [17] and compared the effects of plasma gel and hyaluronic acid (HA), the current gold standard for skin rejuvenation, on human dermal fibroblasts. Plasma gel promoted greater fibroblast proliferation than HA. However, the absence of a control group in that study limits direct comparison with the present findings [19].

According to Miron et al. [24], concerns have been expressed regarding the use of ACs, which are known to inhibit wound healing in platelet concentrates. Their study compared PRP and i-PRF on tissue regeneration in human gingival fibroblasts, using RCFs of 123 g for PRP and 60 g for i-PRF, further reinforcing the LSCC applied in the present study. Both formulations exhibited high biocompatibility and significantly enhanced fibroblast migration and proliferation compared with controls, findings that are consistent with the present results. Moreover, i-PRF induced higher migration, whereas PRP promoted greater cellular proliferation, a pattern similar to that observed here, particularly for G3 and G4. It should be noted that gingival fibroblasts were used in that study, whereas dermal fibroblasts were used in the present investigation. Additionally, PRP showed early growth factor release, while i-PRF demonstrated greater sustained release, which may influence long-term clinical outcomes.

Another study demonstrated the superior regenerative potential of i-PRF compared with PRP in human skin fibroblasts, highlighting the advantages of AC removal from platelet concentrates [25]. Enhanced cell migration was observed in plasma (PRP or PRF) treated groups, corroborating findings from Cho et al. [26]. In the present study, our results indicate that Group G2 exhibited an amplified expression profile for FN1 at 72 h. In contrast, Groups G3 and G4, which showed better performance in viability and migration assays, displayed lower and more stable expression levels over time, not only for FN1, but also for COL1A1.

From a biological perspective, FN1 is typically associated with early extracellular matrix (ECM) formation, while COL1A1 reflects later matrix deposition and tissue remodeling. Therefore, the reduced FN1 expression at 24 h across all treated groups suggests an initial delay in provisional matrix formation. However, the pronounced late upregulation observed in G2 indicates a compensatory or dysregulated ECM response, rather than an efficient and timely remodeling process [27].

This delayed expression pattern in G2 is consistent with its reduced metabolic activity and slower wound closure, suggesting that ECM deposition occurs later and less efficiently. In contrast, G3 and G4 likely promote a more balanced and temporally coordinated ECM remodeling, which may explain their superior performance in migration and viability assays, even without marked overexpression of COL1A1 and FN1 at later time points [28,29]. In addition, in this study, RT-qPCR analysis was designed to provide an initial assessment of the transcriptional response of fibroblasts to different plasma gel formulations, with a particular focus on the temporal regulation of extracellular matrix (ECM)-related genes, such as COL1A1 and FN1. However, future studies would be important, including protein-level validation and matrix deposition analyses, to better establish the functional relevance of the observed gene expression patterns.

Overall, studies have demonstrated the high regenerative potential of PRP, particularly due to its growth factor content, even when compared with plasma gel. Although both platelet concentrates contain substantial amounts of bioactive proteins, growth factor concentrations are usually higher in PRP. Therefore, the platelet-rich plasma fraction located just above the red blood cells should be incorporated into the gel after heating to compensate for growth factor denaturation. This approach was supported by the present findings, as all formulations enhanced fibroblast proliferation [30].

In a histological study evaluating fibroblasts treated with PRP, a significantly lower number of fibroblasts was observed in the control groups compared with treated groups by day 3 [31], a pattern similar to that observed in the present study using plasma gel. Another relevant aspect concerns the immersion protocol of plasma gel in culture medium. The present study followed the study by Anitua et al. [17], who recommended a 24 h immersion period, whereas other studies employed longer periods (72 h) [24,25]. This difference in immersion time may have influenced the magnitude of the observed proliferative effects, and future studies are warranted to clarify this issue.

HE staining demonstrated a higher number of cells in Groups 3 and 4, corroborating the viability and proliferation results obtained after 72 h, without alterations in cell morphology. Similarly, Wang et al. [25] reported no significant morphological differences among fibroblast groups treated with platelet concentrates, reinforcing their biocompatibility. Regarding the wound healing assay, G2 exhibited slower closure, comparable to the control group, further supporting the non-toxic nature of platelet concentrates. However, image analysis in G1 and G2 was partially impaired by the presence of fibrin, particularly at 72 h, in both HE staining and migration assays. It should be noted, however, that we did not include proliferation inhibitors in our scratch assay. This means that our results might have been influenced by cell proliferation in addition to migration.

The interference of fibrin observed in morphological and migration analyses further supports the interpretation that biomaterial-derived artifacts may also affect colorimetric assays, reinforcing the importance of incorporating complementary methodologies such as Neutral Red in experimental designs involving fibrin-based scaffolds [32]. Another limitation of the study was the lack of a detailed biochemical and structural characterization of the resulting plasma gel materials, such as platelet concentration, growth factor content, or fibrin structure. In particular, variations in centrifugation force, the use of ACs, and activation methods can affect the distribution of platelets, leukocytes, and fibrin density, thus modulating the release kinetics of bioactive molecules. Consequently, the observed differences in fibroblast behavior suggest that the groups prepared under low-speed conditions (280 g) and without ACs (Groups G3 and G4) generate a more bioactive framework. Therefore, future studies should incorporate plasma gel characterization. In addition, the use of plasma derived from a single donor represents an important limitation of this study, particularly with respect to statistical power and the generalizability of the findings. Platelet concentrates are known to exhibit substantial inter-individual variability in platelet count, growth factor content, and fibrin architecture, which may significantly influence cellular responses. Therefore, although the present in vitro design allowed strict control of experimental variables and enabled the comparison of centrifugation protocols under standardized conditions, the results should not be interpreted as representative of population-level biological responses. Future studies including multiple donors and larger sample sizes are required to validate the reproducibility of these findings and to strengthen their external validity. It should also be noted that the statistical analyses performed in this study are based on technical replicates within a controlled experimental model, and therefore primarily reflect intra-experimental variability rather than inter-individual variability.

Furthermore, this study was limited to short-term cellular responses and did not evaluate long-term outcomes, such as sustained gene expression, extracellular matrix deposition, or biomaterial degradation. Given that plasma gels are designed for volumizing and biostimulatory applications, future studies investigating these long-term effects are crucial to fully elucidate their clinical performance, stability, and durability over time. Additionally, future studies would benefit from the use of more advanced experimental models, such as co-culture systems or organotypic skin constructs, to better mimic in vivo conditions. Another limitation of the present study is the use of only one plasma donor, which does not consider interindividual variability. Future studies should include more plasma donors to account for biological variability. Moreover, our study design included only two independent experiments with technical replicates, which might reduce the robustness of the statistical analyses. Furthermore, the expression of extracellular matrix genes was evaluated only at the RNA level, without validation at the protein level. Future studies should consider these limitations.

In the present study, Anitua’s protocol [17] was used as a reference for plasma gel preparation. In this protocol, CaCl_2_ is required to activate coagulation due to the presence of ACs in collection tubes. Since collagen is a natural activator of coagulation, we evaluated plasma gel preparation in the absence of CaCl_2_ for both RCFs tested. When G3 was compared with G4, faster wound closure was observed for G3 at early time points (24 h), but after 72 h, both groups exhibited similar closure percentages. These findings indicate that exogenous activation is not required when centrifugation is performed at 210 g for 8 min using tubes without additives.

## 3. Conclusions

Although this is a preclinical, single-donor study, the results demonstrated superior fibroblast behavior in Groups 3 and 4, which employed LSCC (210 g) and tubes without ACs. Plasma gels prepared under these conditions supported human dermal fibroblast viability and migration. Furthermore, activation with CaCl_2_ was not necessary when LSCC and additive-free tubes were used.

However, these results should be interpreted as preliminary due to the in vitro design of the present study. Further studies are required to validate these findings using samples from multiple donors, given the known biological variability in blood-derived products. Moreover, future studies should provide a more comprehensive characterization of plasma gel formulations, not only regarding their cells and growth factors composition, but also considering the rheological properties and microstructural characteristics of the formulations. In addition, investigations under conditions that more closely mimic clinical applications are essential to optimize clinical protocols, understand the process of developing long-lasting volumizing effects and the biostimulatory potential of plasma gels.

## 4. Materials and Methods

### 4.1. Preparation of Plasma Gel

One young, healthy individual, with no relevant medical history and not using any medication, a postgraduate student from the Department of Biological Sciences (FOB/USP/Bauru), served as the volunteer to donate the blood necessary for the development of the experiments.

Four blue-cap tubes containing 3.6 mL of blood were collected from the volunteer into collection tubes containing 0.4 mL of 3.8% sodium citrate (*w*/*v*) as an AC [17]. Another four white-cap plastic tubes (9 mL) without AC were also collected. The samples were centrifuged in a FibrinFUGE25 digital centrifuge (Montserrat, São Paulo, Brazil) with a rotor radius of 65 mm and a fixed angle of 25°, following the protocols described below:

• Group 1 (G1): Blood collected in blue-capped tubes (with 3.8% sodium citrate) was centrifuged at 580 g for 8 min. The PPP (upper ⅔) was collected and heated at 76 °C for 12 min to obtain the precursor gel. The rich plasma (RP) (lower ⅓, F2 fraction) was collected, activated with 10% CaCl_2_ (20 uL/mL), and mixed with the precursor gel in a 1:5 ratio [17].
• Group 2 (G2): Blood collected in blue-capped tubes (with 3.8% sodium citrate) was centrifuged at 580 g for 8 min. The PPP (upper ⅔) was collected and heated at 76 °C for 12 min to form the precursor gel. The RP (lower ⅓, F2 fraction) was collected and mixed with the precursor gel in a 1:5 ratio [17].
• Group 3 (G3): Blood collected in white-capped tubes (without AC) was centrifuged at 210 g for 8 min. The PPP (upper ⅔) was collected and heated at 76 °C for 12 min to obtain the precursor gel. The RP (lower ⅓, F2 fraction) was collected, activated with 10% CaCl2 (20 uL/mL), and mixed with the precursor gel in a 1:5 ratio [16,17].
• Group 4 (G4): Blood collected in white-capped tubes (without AC) was centrifuged at 210 g for 8 min. The PPP (upper ⅔) was collected and heated at 76 °C for 12 min to obtain the precursor gel. The RP (lower ⅓, F2 fraction) was collected and mixed with the precursor gel in a 1:5 ratio [16].
• Group C (control group): Cells cultured only with culture medium, without exposure to plasma gel.

### 4.2. Cell Culture

Primary Human Dermal Fibroblasts (FBH CK 001) (normal human skin fibroblasts; Koamoscience) were donated by Dr. Ana Lúcia T. A. Pinheiro from the University of São Francisco (Research Ethics Committee CAAE 2.493.285). Cells were cultured in Dulbecco’s Modified Eagle Medium (DMEM) supplemented with 10% fetal bovine serum (FBS) and 1% antibiotics under standard culture conditions [17]. The cells were kept at 37 °C in a humidified atmosphere containing 5% CO_2_. Cells between the sixth and eighth passages were used in the experiment. This passage range was selected because primary cells generally preserve their phenotypic stability and biological behavior more reliably during early to intermediate passages [33]. However, in future investigations, it would be recommended to carry out specific validation and characterization of the primary fibroblasts obtained, since these results provide an important basis for the reliability of new discoveries.

After preparation, the plasma gel was incubated for 90 min at 37 °C to allow enough cross-linking. The biomaterial (20%/20 g/100 mL) was then immersed in culture medium and incubated for 72 h at 37 °C to obtain leachates. The resulting conditioned medium (plasma gel leachates) was collected and used for all experiments (G1–G4) [17].

### 4.3. Viability Assay: MTT

Cells were seeded at a density of 2 × 10^4^ cells/well in 96-well plates and allowed to adhere for 24 h. Subsequently, the culture medium was replaced with medium containing 20% plasma gel leachates, according to the respective experimental groups G1, G2, G3, G4 or the control group, and cells were incubated for 72 h under culture conditions (37 °C, 5% CO_2_). After the incubation period, 110 μL of MTT solution (0.5 mg/mL; 3-(4,5-dimethylthiazol-2-yl)-2,5-diphenyltetrazolium bromide) prepared in DMEM without FBS was added to each well and incubated for 4 h at 37 °C. Then the supernatant was removed, and the intracellular insoluble formazan crystals were dissolved in 200 μL of dimethyl sulfoxide (DMSO) per well, followed by gentle agitation for 15 min at room temperature. Absorbance was measured at 550 nm using a microplate reader (Synergy™ Mx, BioTek Instruments Inc., Winooski, VT, USA), and the cell viability was expressed relative to the control group [17,34].

### 4.4. Viability Assay: Crystal Violet

Crystal violet staining was used to assess cell viability by binding to nucleic acids in adherent cells. Cells were plated in 96-well plates using culture medium supplemented with 10% FBS and 20% plasma gel, and maintained for 72 h, as briefly described by Anitua et al. 2018 [17]. After incubation, the culture medium was carefully removed, and 50 μL of 0.05% crystal violet solution was added to each well, followed by incubation for 20 min at room temperature with gentle agitation. The plates were then rinsed with running water to remove excess dye and allowed to air-dry for at least 24 h. To solubilize the retained dye, 200 μL of methanol was added to each well and incubated for 20 min at room temperature. Absorbance was subsequently recorded at 570 nm using a microplate reader (Synergy™ Mx multimode monochromator microplate reader, BioTek Instruments Inc., Winooski, VT, USA) [19,35].

### 4.5. Viability Assay: Neutral Red

Cell viability was additionally assessed using the Neutral Red uptake assay, based on the ability of viable cells to incorporate and retain the supravital dye within lysosomes. Cells were seeded at a density of 2 × 10^4^ cells/well in 96-well plates and allowed to adhere for 24 h. Subsequently, the culture medium was replaced with medium containing 20% plasma gel leachates (according to experimental group: G1-G4), and cells were incubated for 72 h under standard culture conditions (37 °C, 5% CO_2_). After the treatment period, the medium was removed, and 100 μL of Neutral Red solution (40 μg/mL in serum-free DMEM) was added to each well and incubated for 2 h at 37 °C. Cells were then gently washed with phosphate-buffered saline (PBS) to remove excess dye. The incorporated dye was extracted by adding 150 μL of destaining solution (50% ethanol, 49% distilled water, 1% acetic acid) to each well, with gentle shaking for 10 min at room temperature. Absorbance was measured at 540 nm using a microplate reader (Synergy™ Mx, BioTek Instruments Inc., Winooski, VT, USA), and cell viability was expressed as a percentage relative to the control group [36].

### 4.6. Qualitative Analysis Using Hematoxylin-Eosin (HE) Staining

Cells were plated at a density of 3 × 10^4^ cells on 13 mm diameter glass coverslips positioned in 24-well plates. Following cell attachment, the culture medium was replaced with medium supplemented with plasma gel, and the cells were maintained for 72 h. Subsequently, the cells were fixed with 10% neutral buffered formalin for 30 min and rinsed with phosphate-buffered saline (PBS). For morphological evaluation, samples were stained with hematoxylin for 3 min, differentiated using 0.5% acid alcohol, and counterstained with 1% eosin for 2 min. The specimens were then dehydrated in a graded ethanol series, cleared in xylene, and mounted with Entellan^®^. Images were acquired using an Olympus U-TV0.5XC-3 digital camera (Olympus, Tokyo, Japan) coupled to a microscope at 40× magnification [35].

### 4.7. Wound Healing Assay (Closure Test)

Cell migration was evaluated as described by Carvalho et al. [19]. Cells were seeded at a density of 1.5 x 10^5^ in 12-well plates and cultured until confluence (around 96 h). A linear scratch was created using a 1000-μL pipette tip. After washing with PBS, medium containing plasma extracts and 0.5% FBS was added. Images were captured at 0, 24, 48 and 72 h using a phase-contrast microscope connected to the Olympus U-TV0.5XC-3 digital camera (Evident Corporation, Hamburg, Germany), with a 4× objective. Triple-blind observational analyses were performed, and experiments were performed in triplicate. The wound area was quantified using Image J software, and the following equation was used to calculate closure percentage: % closure = [(A0 − A72)/A0] × 100 [34].

### 4.8. RT-qPCR Assay

The expression levels of the extracellular matrix genes collagen type I alpha 1 (COL1A1) and fibronectin (FN1) were evaluated by RT-qPCR, with GAPDH used as the housekeeping gene. Total RNA was isolated using the PureLink RNA Mini Kit (Invitrogen), and RNA quantity and purity were determined using a Take3 Trio plate in an Agilent BioTek^®^ microplate reader. Complementary DNA (cDNA) was synthesized with the High-Capacity cDNA Reverse Transcription Kit (Applied Biosystems). Quantitative PCR reactions were carried out using TaqMan™ Gene Expression Master Mix (Applied Biosystems™) together with specific assays for FN1 (Hs01549976_m1), COL1A1 (Hs0016004_m1), and GAPDH (Hs99999905_m1) on a ViiA™ 7 Real-Time PCR System (Applied Biosystems). Additional details are provided in Supplementary Table S1. The relative transcript levels were determined using the 2^−ΔΔCt^ method, as previously described [37].

### 4.9. Statistical Analysis

All experiments were performed in at least two independent replicates. Statistical analyses were conducted using GraphPad Prism 8 software (GraphPad Software Inc., La Jolla, CA, USA). Data normality was evaluated using the Kolmogorov–Smirnov test. One-way ANOVA followed by Tukey’s post hoc test was used for multiple comparisons, and paired Student’s *t* test was applied for comparisons between two groups. Data are presented as mean ± standard deviation. *p* values < 0.05 were considered statistically significant.

## Data Availability

The datasets generated during and/or analyzed during the current study are available from the corresponding author on reasonable request.

## Supplementary Materials

The following supporting information can be downloaded at: https://www.mdpi.com/article/10.3390/gels12080713/s1, Table S1: TaqMan ® Gene Expression Assays used for RT-qPCR analysis, including target genes and corresponding assay IDs (Applied Biosystems).

## Abbreviations

The following abbreviations are used in this manuscript:

PRP	platelet-rich plasma
PRF	platelet-rich fibrin
i-PRF	injectable platelet-rich fibrin
PPP	Platelet-poor plasma
ACs	anticoagulants
RPM	revolutions per minute
LSCC	Low-Speed Centrifugation Concept
ECM	extracellular matrix
RCF	relative centrifugal force
