# Supplementary material for: Effect of Plasma Gel on Viability, Proliferation and Migration of Dermal Human Fibroblasts In Vitro
Source: Gels. 2026 Aug 12;12(8):713. doi: 10.3390/gels12080713 (PMC13512267; doi:10.3390/gels12080713)
Supplement: Supplementary file 1 [file gels-12-00713-s001.zip › gels-4433748-supplementary.pdf]

## Supplementary Section

| **TaqMan® Gene Expression Assays** | **Assay ID** |
|---|---|
| GAPDH | Hs99999905_m1 |
| FN1 | Hs01549976_m1 |
| COL1A1 | Hs00164004_m1 |

**Supplementary Table S1.** TaqMan® Gene Expression Assays used for RT-qPCR analysis, including target genes and corresponding assay IDs (Applied Biosystems). GAPDH was used as the endogenous control for normalization. Relative gene expression was calculated using the $2^{\wedge -\Delta\Delta Ct}$ method.
